# Five views on the foundations of data science

**DOI:** 10.1016/j.patter.2026.101566

**Published:** 2026-06-12

**Authors:** Bruce S. Kristal, Melissa D. McCradden, Jeyan Thiyagalingam, Zijun Zhang, Carole Goble

## Abstract

In this People of Data piece, our advisory board members select and discuss papers that they consider to be foundational to data science.

## Main text

Data science is, by its nature, an interdisciplinary field of research, incorporating techniques from multiple other fields, including statistics, complexity research, and computer science. Its methods are applied to a range of scientific domains wherever complex data exist, and the field itself generates challenging questions that drive academic enquiries in ethics, philosophy, science policy, and well beyond. To explore this breadth, we asked five of our advisory board members to select and discuss papers that they consider “foundational” to the field of data science.

The selected papers highlight the diverse foundations of the field, and only one paper is mentioned by more than one board member.^1^ The papers were published at journals, in conference proceedings, as book chapters, and even in one case, simply on a departmental website.^2^ While most were published in the last 20 years, the oldest paper is from the 18^th^ century, demonstrating the deep roots of the field and the dangers of taking a narrow, short-term perspective when considering scientific impact.

We invite you to explore these different perspectives on the foundations of data science and these notable scientific works.

### Bruce Kristal

Data science may be broadly defined as the scientific study of how to optimize the use of *data*; foundational papers in data science accordingly open new vistas for thinking about what data are or how to use them to address real-world problems.

*One aspect of data science focuses on identifying approaches to use data to determine what decision to make, how much confidence to have in a belief, and/or when to change a belief or decision.* Price’s presentation of Bayes’ work lays out what becomes Bayesian analysis.^3^ Now over 250 years old, this work still lies at the heart of how we think about clinical data and decision-making, and variants on its approach cross domain boundaries. I first ran into its (shocking!) implications in graduate school as a virologist (notably, without ever hearing the word “Bayes”), when it was revealed that, if a person testing positive for HIV infection was not a member of a high-risk group, they were more likely *not* to be infected with HIV than to be infected. While today’s formal statement involves priors and posterior probabilities, it is an alternative notation (often attributed to Pierre-Simon Laplace) that I’ve found more intuitive—and more directly relevant for data science. Arguably, this one short equation suggests that we rethink the sole emphasis on frequentist framing central to the education of most scientists today.

$$P\left(H|D\right)=\frac{P\left(D|H\right)P\left(H\right)}{P\left(D\right)}$$

*P*(*H*│*D*)—what is the probability that the hypothesis is correct given *these data*? This flips the usual framing: we no longer are asking how likely the data are—*the data now becomes central truth*—we now ask how likely is the hypothesis/model given the observed data.
*P*(*D*│*H*)—what is the probability that we would see *these data* given the hypothesis? There are no null hypotheses and no assumptions; instead, at least in ideal cases, this term can itself be directly estimated from *data* (e.g., test sensitivity).
*P*(*H*)—what is our estimate that the hypothesis was correct before the current *data* (the prior)? In predictive risk questions—for example, *P*(*H*) for “this person will have ovarian cancer in five years”—the prior becomes progressively sharper as we condition from a population to women in that population to women over 20 years old and so on. These refinements can be entered directly or through a Bayesian lens—again using *data* to progressively strengthen this term.
*P*(*D*)—what is the probability that we see *these data* regardless of whether the hypothesis is correct? Again, the *data* themselves take center stage, and Bayes’ rule provides a persistent guide for making best use of them.

The centrality of the *context of data* and the problem under study is revealed by Wolpert and Macready’s development of the “no free lunch theorems” in computer science (e.g., Wolpert and Macready ^4^). The take-home lesson is straight-forward and central to data science: context matters. To conduct a rigorous and powerful analysis of a dataset, we must always consider the data in the context of both its origins and the specific problems of interest.

Another aspect of data science focuses on how we can best learn what the data have to tell us. Breiman’s “two cultures” paper,^5^ published in 2001 as “informatics analysis” accelerated, draws a stark line between classical statistical modeling and what we would now call informatics approaches. In the classical view, statistics asks whether the data observed fit a mathematically convenient, pre-specified model (i.e., P(D│H)), often framed in terms of a null hypothesis. Inherently, formal frequentist statistics rest on the assumption that it understands reality well enough to pre-identify the ideal model to test. Breiman, the originator of the random forest algorithm, argues convincingly to let the data speak.

A fourth aspect of data science focuses on how we can best explore data, and, arguably more importantly, do so while minimizing over-fitting in its various forms. One “super-power” of informatics approaches lies in their ability to “see” information in the data that is wholly unanticipated; indeed, this is inherently central to Breiman’s argument. Such power is already present in relatively basic approaches (e.g., principal-component analysis and similar projection techniques), expands further in nonlinear methods and machine learning (e.g., CART and random forests), and further increases in neural networks and today’s AI approaches. The challenge, then, is deciding what we can trust. Statistics has long recognized the problems posed by multiple comparisons, and developed approaches such as ANOVA with post hoc testing, Bonferroni/Sidak corrections, and false-discovery-rate control. The problem is magnified in informatics, given the rapidly spiraling increase in variables and inputs when one considers interactions and coefficients. There are technical approaches to help address these concerns (e.g., permutations and out-of-bag test sets), but best practice begins by proactive consideration of this complexity during initial planning and design. This is the message of Gelman and Loken’s “garden of forking paths” paper^2^ published in 2013 just as the concepts of “data science,” “big data,” and “AI” accelerated their impact on the modern data-based world. “Forking paths” focuses on the importance of pre-specification and understanding how seemingly “correct” data-based adjustments in analysis (and in presentation and interpretation)—and the associated degrees of freedom—can readily cause overfitting, even when using only basic statistics. Following the data offers critical opportunities to learn and to develop testable hypotheses, but keeping the “forking paths” required to reach these revelations in mind is equally critical.

Data science places data as the central target of investigation; these four papers represent landmarks in understanding the power of data, in context, to change opinion and to provide unexpected insights and signals to probe nature, and they highlight the danger of losing sight of the power of modern approaches to find signals at any cost.

### Melissa D. McCradden

#### Data are people and people are data

The first thing you need to understand is that people are data and data are people. Bioethics and ethical reflection are grounded primarily in the inherent dignity and respect each person is due. Deb Raji reflects on the insensitivity and distance that data scientists can embody in the statistical processes they apply to data, forgetting the humans they represent: “In data science, there is the frustrating insistence that our counting is neutral and lacks consequences.”^6^ Raji’s examples stem from the pandemic, but their implications are broader. Routinely collected clinical data, for example, represents moments of patients’ lives where they are at their most vulnerable, most worried, and positioned to have their lives permanently changed.

This lesson is illuminated nicely by Maori knowledge holders, who conceptualize data as “taonga”—meaning something precious or significant.^7^ Under this conceptualization, the obligations of all those who hold data is less about whether it is de-identified, anonymous, or privacy protected and more about the relationality inherent within taonga. For example, considering the value, level of trust, originality, and nature of the application enhances traditional data quality assessments like FAIR.^8^ If data scientists moved beyond data as a commodity and toward one of custodians of taonga, we might improve the ethical culture around data practices.

#### Fairness is not a numbers game

“Bias” has perhaps become the most useless word in data science for describing anything of value. As statisticians often note, “bias” has many meanings that range from benign statistical patterns to algorithmic racism. Abeba Birhane^9^ offers a justice-based framing of how statistical biases reflect real systematic patterns of differential treatment and problematizes how data science encourages “solutionism” under the backdrop of deeply complex social conditions.

The strong temptation to “fix” problems like bias stems from a genuine moral motivation among data scientists. Without dismissing this moral aim altogether, Birhane notes that we must unpack how this framing assumes a single “correct” answer: the implication being that fixing bias means finding the correct (objective) pattern. First, we must recognize that there are no neutral choices. The choices of whether to not look for bias, to try to make an unbiased algorithm, or to simply model patterns apparent in the data are all choices that reflect values. These are not choices that should be made alone. Birhane emphasizes that isolated, binary choices lead to an impoverished understanding of the system one is trying to develop. Rather, she highlights the importance of thinking in relational terms to a rich understanding of technological systems; a way of thinking that necessitates seeking diverse views and knowledges.

Linked to the first lesson above, it is easy to forget in all the conference presentations and journal papers that data are people and the very real consequences of algorithmic discrimination. Part of the problem is that “algorithmic bias” is a misnomer in two ways: one, bias is often present because of underlying patterns of clinically insignificant differences in care as a function of demographics; two, when we neglect to measure and mitigate fairness threats, we are actively choosing *against* safety and equality in AI. While some decision-making may be beyond the remit of most data scientists, all data scientists can nonetheless advocate for fairness analyses as part of a core commitment to professional and responsible data science practices.

Birhane advocates for *understanding* over mere prediction to improve ethical data science practices, which means knowing where to go to improve understanding. In Justice, Equity, Fairness, and Anti-Bias (JustEFAB^10^), we offer a framework for data scientists to work through these complex problems and address algorithmic fairness concerns at every stage of the AI life cycle.

#### The data we need isn’t the data we have (right now)

There is still a major gap between the computational ability to make predictions and the use of those predictions to bring actual benefit to patients and healthcare systems. One of the reasons is that the data we are using to train models are often not fit-for-purpose. Alex John London^11^ takes on the “knowledge ecosystem” represented by our current system’s health data and demonstrates how fundamental problems with how we capture data about health and medicine will inevitably limit the social value of that data. He points to a recent systematic review that demonstrated that two-fifths of randomized clinical trials evaluating models with good performance in an observational setting failed to benefit patients compared with standard care.^12^

The first step to moving toward a “healthy knowledge ecosystem”^11^ would be to ensure our translation process prioritizes information-gathering practices that lead to socially valuable knowledge about an AI system. If we understand that prediction is not benefit, we can augment model evaluation with additional metrics that are related to clinical utility, a notion we have called the “intervention ensemble” for healthcare machine learning.^13^ Furthermore, we can and should include additional measurements that relate to value in all its forms. For example, an accurate model with good clinical utility will not benefit anyone if no one can pay for it or if there are not enough beds or staff to care for the patients whose access has increased. Avoiding deployment for deployment’s sake therefore encourages two practices: (1) creation of models that are aimed at clinically valuable tasks rather than convenient labels and (2) evaluation of the intervention ensemble that establishes overall benefit rather than just prospective model performance.

### Jeyan Thiyagalingam

#### Attention is all you need

The introduction of the transformer architecture marked a major shift in how models learn from data.^1^ By replacing recurrence and convolution with attention-based mechanisms, the paper demonstrated that long-range dependencies could be captured more efficiently and flexibly. This architectural change not only improved performance on core sequence modeling tasks but also enabled rapid scaling to very large models. The transformer has since become the foundation for a wide range of applications, including language modeling, protein structure prediction, weather forecasting, and a range of scientific simulations. Its modularity and ability to parallelize computations across tokens allowed the community to train very large models and explore data scaling in ways that were previously impossible. Much of today’s progress in generative modeling and scientific machine learning and even on hardware architectural space builds, directly or indirectly, on the ideas introduced in this paper. For these reasons, it has become one of the most influential works in modern machine learning and a key part of the methodological foundation for many data-driven scientific workflows.

#### Neural operator learning

Neural operator methods introduced an approach for learning mappings between function spaces rather than learning pointwise mappings or local predictors.^14^ This shift has been influential across scientific domains where underlying systems are governed by partial differential equations or operator-based relationships. The Fourier Neural Operator (FNO) demonstrated that global operators could be learned efficiently by parameterizing them in Fourier space, enabling fast inference and reliable generalization across different discretizations and resolutions.^15^ This represented a substantial departure from conventional surrogate models trained on fixed grids or domains. Neural operators now support many efforts in data-driven simulation, climate modeling, fluid dynamics, and materials science, where they offer significant speedups over numerical solvers while producing outputs that reflect coherent spatial and temporal structure. As scientific disciplines increasingly integrate machine learning into modeling pipelines, operator learning provides a bridge between data-driven approaches and traditional simulation-based approaches. The conceptual clarity and practical effectiveness of FNO make it a foundational contribution to the rapidly growing area of scientific machine learning.

#### Variational auto-encoders

The variational auto-encoder (VAE) provided a scalable and principled framework for learning latent variable models via variational inference.^16^ The key contribution was making probabilistic generative modeling accessible through simple neural network components and stochastic gradient optimization. This opened the door for several subfields within generative modeling, from modeling uncertainty to disentangled representations and to continuous latent spaces in high-dimensional data. VAEs established many of the ideas that underpin today’s generative models, including diffusion models since both rely on latent variable formulations and tractable approximate likelihoods. For scientific applications, VAEs offered a way to learn generative priors, compress complex datasets, and explore structured latent manifolds that reflect underlying physical or biological variation. Their flexibility has led to use cases in microscopy, materials discovery, molecular design, and medical imaging. Although subsequent models have improved various aspects and shortcomings of the original model, the conceptual foundations introduced by VAEs remain central to generative modeling and representation learning.

### Zijun Zhang

Tracing back fundamental works that have generated profound impacts in machine learning and data science fields, we might immediately recall the golden three years, 2015–2017. LeCun et al.’s 2015 review^17^ announced the start of a new era, with modeling methods moving from shallow to deep learning paradigms. Next year, He et al. presented ResNet, a milestone deep network architecture design.^18^ In 2017, “Attention is All You Need” by Vaswani et al. offered us a preview of the coming dominance of transformer models.^1^ The community was excited about these conceptual advances, which have elevated the empirical application performance of data-driven methods and raised solutions to a new level. Yet, we are also well aware of the long-lasting headaches created by data-driven methods and models, including problems related to trustworthiness and generalizability. Focusing on these areas, I would like to highlight three works that I would personally consider as key milestones.

#### Domain adaptation

Ganin et al. presented a representation learning method for domain adaptation, which focused on deriving features that cannot discriminate between the training and test domains.^19^ Such a concept has served as the foundation for the many learning scheme developments for domain adaptation and generalization as well as their applications, which basically aimed to learn invariant features, source-target-domain aligned features, knowledge transferrable features, etc. The characteristics of latent features learned can be leveraged to justify the generalizability of data-driven models in cross-domain applications.

#### Shapley additive explanations

Lundberg and Lee presented Shapley additive explanations (SHAP) in 2017, an interpretability framework grounded in cooperative game theory that assigns values to features based on their contribution to model predictions.^20^ SHAP is considered to be a model-agnostic approach and can be applied to help interpret the performance of data-driven models developed by various machine learning algorithms. SHAP provides both global and local explanations of data-driven models, making it a versatile tool for model interpretation in various applications.

#### Physics-informed neural networks

The work presented by Raissi et al. in 2019 is an important milestone in the development of physics-informed neural networks (PINNs).^21^ The generic version of PINNs involves a framework integrating neural networks with physical principles to solve complex problems involving nonlinear partial differential equations (PDEs). Compared with classical neural networks, PINNs’ unique concept of integrating physical principles into their architecture helps improve model generalization and trustworthiness, leading to wide adoption in many of the latest science-focused AI studies.

In summary, the aforementioned three works laid a conceptual and theoretical foundation that paved the way for follow-up research efforts studying more advanced techniques and solutions, thereby enhancing the generalizability and trustworthiness of data-driven techniques and solutions.

### Carole Goble

#### What *are* the FAIR principles for scientific data?

The importance of universal and persistent access to well-described data adhering to community standards has been a cornerstone of data science for decades—certainly since the start of my career in the 1980s. Recognition of the concept of Findable, Accessible, Interoperable, and Reusable data by all stakeholders—from researchers to funders and data stewards to publishers, academia, and industry—has escalated in the past decade with the publication of the “The FAIR Guiding Principles for scientific data management and stewardship” in 2016.^8^ Although this is one of the most cited papers in the field, it has also become so seminal that one wonders if those citing it have read it. The paper emphasizes enhancing the *ability of machines to automatically find and use data* in addition to humans, arising from contemporary work on automated data analytics using APIs and computational workflows in the life sciences by many of the authors (including me!). Ten years later, in the era of AI-driven data science and policies of national data sovereignty, the availability of licensed machine actionable metadata and attributable provenance is more critical than ever. But let’s be clear, FAIR is just the first step on the rung of data AI-readiness—as Clark et al. have made clear in an influential pre-print on the Bridge2AI recommendations.^22^

#### FAIR data are not free, need not be open, and are everyone’s responsibility

FAIR is not the same as free—it bears a cost to providers and users, and success requires a socio-technical, holistic understanding of the environment knowledge transfer operates in. “From Data Creator to Data Reuser: Distance Matters” by Borgman and Groth offers recommendations for data creators, data reusers, data archivists, and funding agencies on how to make data sharing and reuse more effective and less burdensome through a framing of the “distance” between data creators and prospective reusers.^23^ Instead of focusing on data sharing, the paper asks who might reuse data, how, for what purposes, and when, proposing six dimensions of distance that influence the ability to transfer knowledge effectively: domain, methods, collaboration, curation, purposes, and time and temporality.

#### Checking for data FAIRness requires automated assistance not draconian judgment

From a technical and pragmatic perspective, how do we know if a data resource is FAIR? Numerous efforts by communities and international organizations (CODATA, GO-FAIR, RDA, etc.) have grappled with this issue, given that FAIR is a spectrum. It is an explicit contract between a data creator and data reuser rather than one universal set of metrics to be judged against. Enabling automated assessment—or rather now we would say *automated assistance*—is a hot topic. An automated solution for measuring the progress toward FAIR research data by Devaraju and Huber in *Patterns* was one of the first papers to describe the translation of the FAIR data principles into measurable metrics and the application of the metrics in evaluating FAIR compliance of research data.^24^ It introduces a still widely used open-source tool, F-UJI, but more importantly, it examines each principle in detail and the challenges of practically accessing and interpreting the metadata at scale and across the data universe. No one said it was going to be easy!

## Acknowledgments

BS.K. declares that his contribution to this study was supported in part by the USDA Agricultural Research Service under Cooperative Agreement 58-8050-3-003. Any opinions, findings, conclusions, or recommendations expressed in this publication are those of the authors and do not necessarily reflect the views of the USDA. J.T. would like to acknowledge that his contribution to this study was partially supported by the AI for Realistic Science 2.0 through UKRI’s ISPF funding.

## Declaration of interests

B.S.K., M.D.M., J.T., Z.Z., and C.G. sit on the *Patterns* advisory board.
